# What predicts citation counts and translational impact in headache research? A machine learning analysis

**DOI:** 10.1177/03331024241251488

**Published:** 2024-05-01

**Authors:** Antonios Danelakis, Helge Langseth, Parashkev Nachev, Amy Nelson, Marte-Helene Bjørk, Manjit S. Matharu, Erling Tronvik, Arne May, Anker Stubberud

**Affiliations:** 1 NorHead Norwegian Centre for Headache Research, Trondheim, Norway; 2 Department of Computer Science, NTNU Norwegian University of Science and Technology, Trondheim, Norway; 3 High Dimensional Neurology Group, UCL Queen Square Institute of Neurology, University College London, London, UK; 4 Department of Clinical Medicine, University of Bergen, Bergen, Norway; 5 Department of Neurology, Haukeland University Hospital, Bergen, Norway; 6 Headache and Facial Pain Group, UCL Queen Square Institute of Neurology and National Hospital for Neurology and Neurosurgery, London, UK; 7 Department of Neuromedicine and Movement Sciences, NTNU Norwegian University of Science and Technology, Trondheim, Norway; 8 Department of Systems Neuroscience, University Medical Center Hamburg-Eppendorf, Hamburg, Germany

**Keywords:** Artificial intelligence, prediction, translational, deep learning, neural networks

## Abstract

**Background:**

We aimed to develop the first machine learning models to predict citation counts and the translational impact, defined as inclusion in guidelines or policy documents, of headache research, and assess which factors are most predictive.

**Methods:**

Bibliometric data and the titles, abstracts, and keywords from 8600 publications in three headache-oriented journals from their inception to 31 December 2017 were used. A series of machine learning models were implemented to predict three classes of 5-year citation count intervals (0–5, 6–14 and, >14 citations); and the translational impact of a publication. Models were evaluated out-of-sample with area under the receiver operating characteristics curve (AUC).

**Results:**

The top performing gradient boosting model predicted correct citation count class with an out-of-sample AUC of 0.81. Bibliometric data such as page count, number of references, first and last author citation counts and h-index were among the most important predictors. Prediction of translational impact worked optimally when including both bibliometric data and information from the title, abstract and keywords, reaching an out-of-sample AUC of 0.71 for the top performing random forest model.

**Conclusion:**

Citation counts are best predicted by bibliometric data, while models incorporating both bibliometric data and publication content identifies the translational impact of headache research.

## Background

The headache research literature is rapidly expanding (1), yet the importance and influence of individual scientific works can be difficult to measure. Citation counts, impact factor and h-index are common metrics of research performance (2). Such metrics traditionally drive research funding, recruitment and indicate the importance of publications (3). It is therefore of interest for both funders, editors and researchers to be able to predict future citation counts of a publication.

Recent advances in artificial intelligence have led to a rapidly expanding body of literature investigating and developing machine learning models that can predict citation counts (4). Predictive citation count models are typically built and trained using bibliometric data such as information about the publishing journal, publication meta-data (e.g. number of authors, word count and number of references), and author and affiliation information, including their publication and citation counts (5,6).

On the other hand, the use of citation counts as a sole metric of research performance has been critiqued as it does not necessarily reflect the real-world output effect of the research (3,7,8). Therefore, models of translational impact, measured as a scientific work’s inclusion in guidelines and policy documents, have recently been developed, with astonishingly high accuracy (9–11). Such models help elucidate the importance of scientific publications beyond the citation count.

At present there exist no domain-specific citation count prediction models for headache research publications, and no established method of evaluating the scientific and medical impact of headache research. The aim of this study was to develop and evaluate the first ever machine learning models for accurately predicting citation counts and the real-world translational impact of headache research papers; and assess which factors are most predictive.

## Methods

In this machine learning study, we used a dataset consisting of all publications from the three headache research journals with the highest impact factor (see below) to develop predictive models of citation counts and translational impact. We followed established guidelines for developing and reporting predictive machine learning models in biomedical research (12) and the Transparent Reporting of a Multivariable Prediction Model for Individual Prognosis or Diagnosis (TRIPOD) Statement (13).

### Data sources and data management

Data used for the analyses was downloaded from Scopus on 10 November 2023. All publications in the headache-oriented journals *Cephalalgia* (e-ISSN: 1468–2982), *Headache: The Journal of Head and Face Pain* (e-ISSN: 1526–4610), and the *Journal of Headache and Pain* (e-ISSN: 1129–2377) were collected from their inception, that is 1981, 1961, 2004 respectively, until 31 December 2017. We aimed to estimate 5-year citation counts, therefore, publications dated after 2017 were not included in the dataset as their relatively lower lifespan would give biased low citation counts. Twenty-eight bibliometric features (variables used for machine learning) were captured for each publication. Important features included: bibliographic meta-data such as page count, number of references, number of authors; first and last author information such as h-index, publication count, and citation count at the time of data download; journal information such as impact factor and immediacy index at the time of data download. To account for varying number of authors, only information on first and last author were captured. In cases where there was only one author, that individual was considered the first and last author. Authors were defined as annotated in Scopus. In addition, Scopus categorizes publications as articles, conference papers, editorials, errata, letters, notes, reviews, and short surveys, and these categories were used to define publication type. The full list of bibliometric features can be found in Online Supplementary Table 1. As machine learning models cannot intuitively handle and interpret text information, each publication’s title, abstract and keywords were converted to numerical vector embeddings using the state-of-the-art Natural language processing (NLP) models Doc2Vec (14), SpaCy (15), and BioBERT (16). Doc2Vec is a document-level model which utilizes neural networks in order to convert an entire set of sentences or a paragraph into numerical vectors. SpaCy is a python package which recruits neural network models that learn from large word corpora to produce numerical vectors. In our case, the large English pipeline model (en_core_web_lg version 3.7.0) was used. BioBERT is also a neural network word representation model that is a trained language model that is further pre-trained on biomedical data collected from PubMed. All of these NLP methods convert the text to a list of numbers that is interpretable by the machine learning models for further analyses. The NLP method with the best prediction accuracy was used in the final model. Data samples with any missing feature information were removed from the dataset. We chose not to impute data as many features had a near infinite number of unique variables (e.g. author affiliations) which would not allow for accurate imputation. Finally, the data was scaled with a Min-Max scaler so that each feature was represented on a uniform range from 0 to 1.

The outcome (label) was defined as number of citations five years after publication. The publications were divided into three classes, defined as three equal-sized quantiles when ordering the publications by their 5-year citation counts. The classes were termed ‘few citations’, ‘some citations’, and ‘many citations’, respectively. The reason for stratifying the label into three classes was that citation counts are usually highly skewed (most publications have few citations, and only a few have very many citations) which makes machine learning regression models (i.e. predicting the exact number of citation counts) inaccurate when the sample size is relatively low (17,18).

Translational impact was defined as a publication’s inclusion in a guideline or policy document using Wellcome Reach (Wellcome Trust; https://github.com/wellcometrust/reach). The Wellcome Reach software extracts the reference lists from documents on the websites of the World Health Organization, UNICEF, Médecins Sans Frontiers, United Kingdom Government, United Kingdom Parliament, and National Institute of Clinical Excellence to provide a dataset of publications included in guideline and policy documents. Publications in the headache journals were matched to the Wellcome Reach dataset using the digital object identifiers and titles to identify which were included in guidelines and policy documents.

To ensure generalizability and avoid overfitting, the dataset was conventionally split into training, validation, and test subsets. The dataset was split into a training set, a validation set and a test set in the ratio 81:9:10 in a randomized fashion. The test set was kept unseen during model training. The top performing model in the validation set was used for final out-of-sample evaluation in the test set.

### Predictive modelling

We trained a series of standard machine learning models to predict the citation counts. Support Vector Machines, k-Nearest Neighbors, Random Forest, Decision Trees, Gradient Boosting, Ada Boosting, Multiple Layer Perceptron and the TabNet (19) deep learning architecture, were evaluated. All of these are standard machine learning techniques used for classification. The performance of all models on the training set was evaluated by stratified 10-fold cross validation using mean micro-averaged accuracy and the mean of the micro-averaged one-vs-rest area under the receiver operating characteristics curve (AUC) (20). AUC is a score between 0 and 1. An AUC of 0.5 corresponds to classification by chance, AUCs above 0.7–0.8 are considered good, and AUCs above 0.8–0.9 are considered excellent. The most promising model was chosen for further optimization where model hyperparameters were optimized using a grid search (Online Supplementary table 3). Next, feature selection was performed by calculating the Pearson correlation between each feature and the citation count. Features with a correlation coefficient absolute value below 0.5 were removed from the dataset. The top performing model during training and validation was evaluated on the test set by AUC and accuracy.

The same modelling strategy was used to predict translational impact (inclusion in a guideline or policy document). Since this outcome was dichotomous, simple two-class AUC and balanced accuracy were used as scoring metrics. Since this outcome was highly imbalanced (only two of every 100 publications were included in guideline and policy documents) a series of resampling strategies, including undersampling, oversampling, synthetic minority oversampling technique and conformal prediction (21) were evaluated. In addition, classifier-inherent balancing techniques, such as equal-class weighting for the random forest and boosting models, were implemented wherever available.

To evaluate the impact of bibliometric data versus text-based publication content (title, abstract and keyword embeddings) we conducted two ablation analyses for both the citation count prediction and the translational impact prediction. Ablation refers to a method to evaluate machine learning models by removing cetain input features and assessing its impact on the performance of the model. The first ablation analysis excluded all text-based features and included only bibliometric data. The second ablation analysis used only text-based features without any bibliometric data. The modelling strategy for the ablation analyses was the same as for the complete dataset. In addition, to address any potential bias from features captured at the time of the Scopus search, we conducted a sensitivity analysis where first and last author citation count, citations per article and h-index were excluded.

For the top performing citation count model, we constructed SHAP (Shapley Additive exPlanations) plots. SHAP uses Shapley values to explain machine learning model predictions, by assigning each feature an importance value. This enables interpretation of how each feature contributes towards a particular prediction and allows visualization of how each feature contributes positively or negatively towards the prediction. It also enables ranking of the different features from most important to least important. For the top performing translational impact model, we constructed an aggregate SHAP summary plot to visualize the relative impact of pooled bibliometric features versus pooled text-based features. We also created a word cloud of the words used in the title and keywords of the ‘many citations’ strata.

Summary statistics were calculated as means with standard deviations (SD) and medians with inter-quartile ranges (IQR) according to distribution. Normality assumptions were based on visual inspection of histograms. No hypothesis tests were made. All analyses were done with Python 3.10 (Python Software Foundation) with the following open-source packages: Pandas 1.5.3, Scikit-learn 1.3.0, Matplotlib 3.7.0, WordCloud 1.9.2, SHAP 0.42.1, pybliometrics 3.4.0 and Gensim 4.3.0.

This research was purely bibliometric without involvement of patients and thus ethical approval and patient consent was not deemed necessary.

## Results

**Figure 1:**
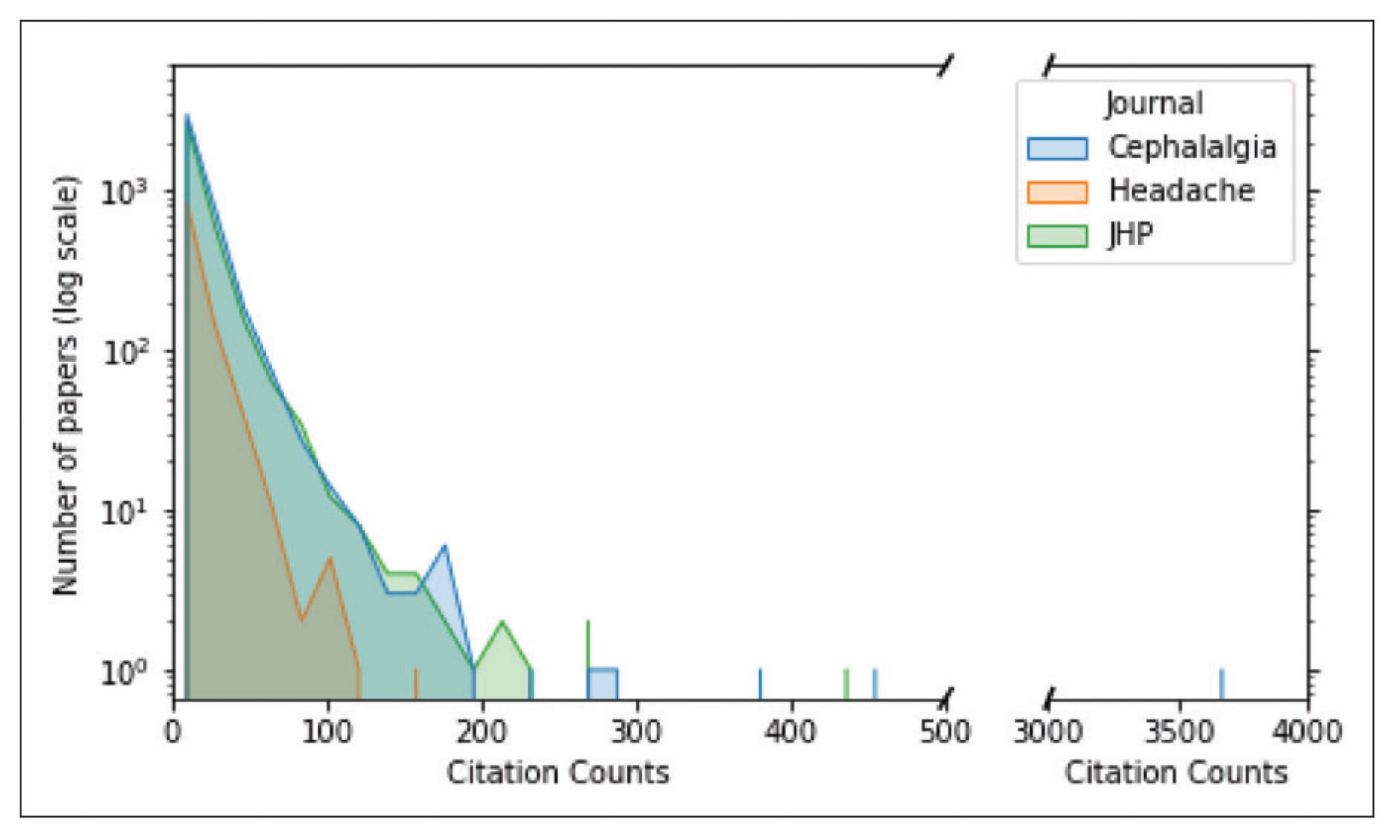
Distribution plot of number of publications per citation count. A distribution plot showing the number of publications with a given 5-year citation counts for three headache journals from their inception to 31 December 2017. The y-axis is on a logarithmic scale. The blue hue represents publications in *Cephalalgia*, the orange hue represents publications in *Headache*, and the green hue represents publications in *Journal of Headache and Pain*. Note that the distribution is skewed, where most publications have few citations, and only a few have many citations (i.e. >200).

A total of 14,279 publications were identified through the Scopus search, but 5679 publications were omitted due to missing data. The remaining 8600 publications were eligible and available for the predictive modelling (Online Supplementary Table 2). Among these, 6481 were original publications, and 2119 were editorials, errata, letters, notes, reviews, short surveys, or conference papers. The quantile classes were nearly equal where 2867 of the total number of publications had few citations (mean number of citations = 1.8 ± 1.6; median = 2, IQR = 0 to 3, range = 0–5), 2867 had some citations (mean number of citations = 9.0 ± 2.7; median = 9, IQR 7 to 11, range 6–14), and 2866 had many citations (mean number of citations = 33.8 ± 14.0; median = 25, IQR = 18 to 36, range >14). Figure 1 illustrates the number of publications per citation count for three journals.

### Citation prediction modelling

Table 1 shows training performance for all evaluated citation count models and the impact of different NLP strategies before hyperparameter optimization. Doc2Vec showed the most reliable and accurate prediction results during training and was chosen as NLP method. The Gradient Boosting classifier displayed the best performance during training with a cross-validated AUC of 0.71 (SD = 0.025) mean training accuracy of 0.52 (SD = 0.024), and a validation AUC of 0.71. Feature selection resulted in the removal of journal volume, issue, start and end pages, year, funding, open access and title length. After feature selection and hyperparameter optimization, the results were further improved, achieving a cross-validated AUC of 0.76 (SD = 0.016), a mean accuracy of 0.56 (SD = 0.023), and validation AUC of 0.76. Out-of-sample test set performance for the optimized Gradient Boosting classifier was 0.78 AUC and an accuracy of 0.58.

**Figure 2:**
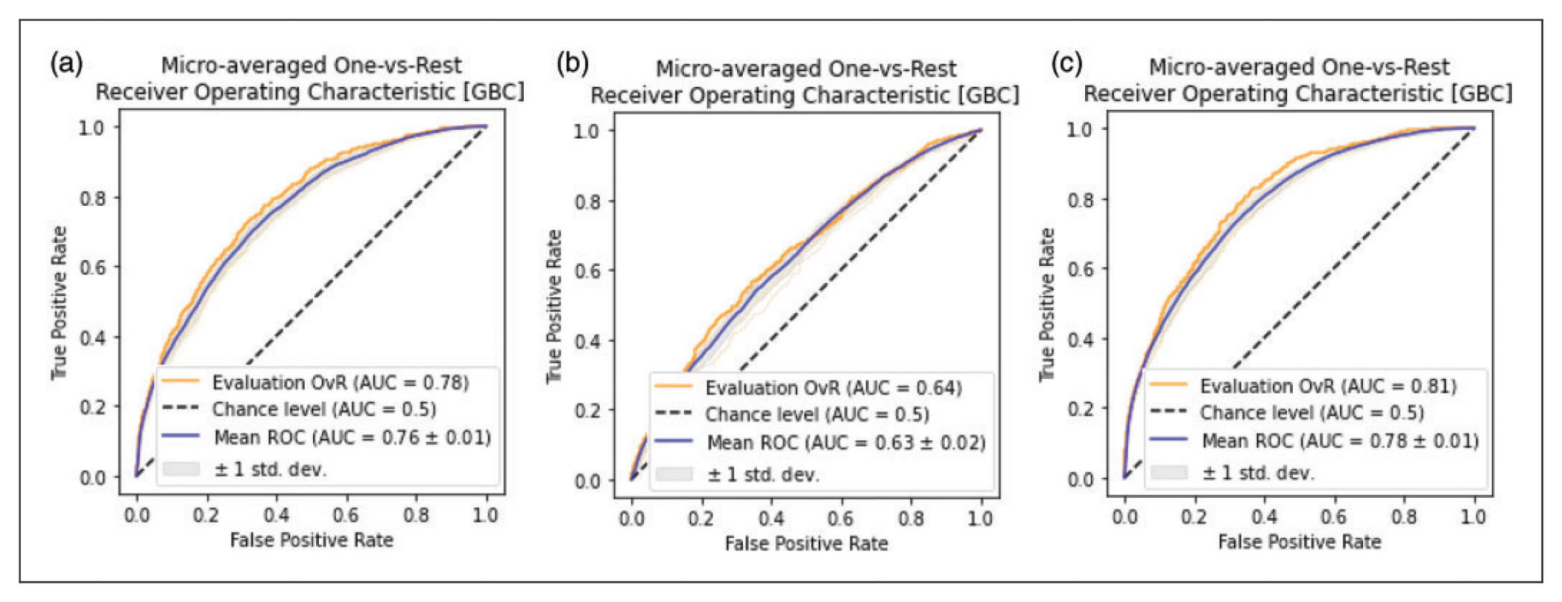
ROC plots of optimized Gradient Boosting model on citation count prediction. ROC plots showing mean training performance (blue line) with 1 standard deviation (gray shaded area) and out-of-sample test set performance (orange line) for the optimal Gradient Boosting citation count model. AUCs are calculated as the micro-averaged One-vs-Rest. (a) Model performance using both bibliometric and text-based features. (b) Model performance using only text-based features. (c) Model performance using only bibliometric features. The highest out-of-sample test set performance is achieved in the bibliometric ablation model (c). ROC = Receiver operating characteristics curve; AUC = Area under curve; OvR = One-vs-Rest.

In the ablation analyses using only text-based features, there was a decrease in accuracy with the Gradient Boosting classifier achieving an AUC of 0.64 and an accuracy of 0.45 in the test set. The ablation analysis including only bibliometric features resulted in a test set AUC of 0.81 and an accuracy of 0.60. Receiver operating characteristics curves for the ablation analyses are shown in Figure 2. The sensitivity analysis resulted in an AUC of 0.78 and an accuracy of 0.59 (Online Supplementary Figure 1).

**Figure 3:**
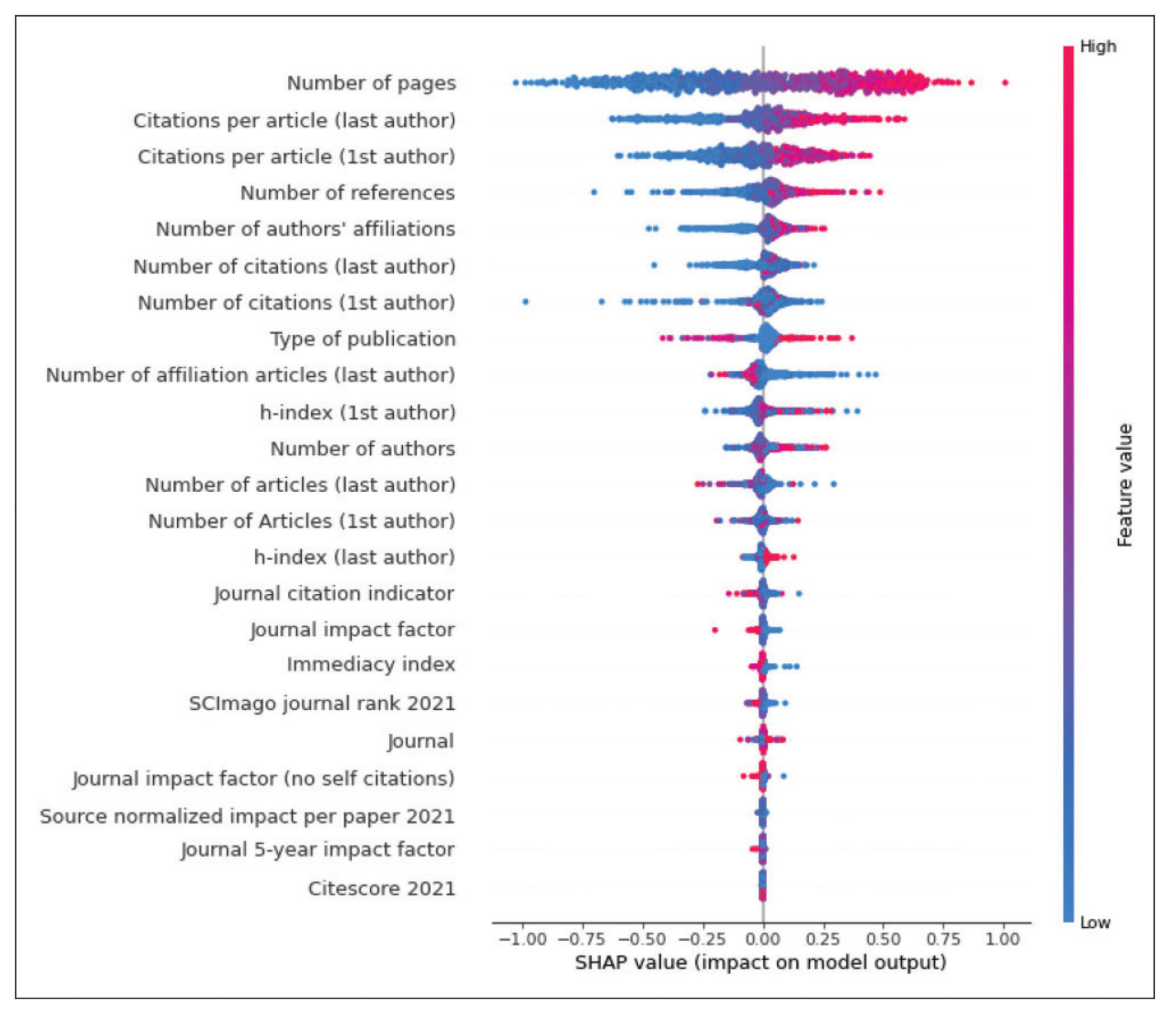
SHAP summary plot for top (bibliometric ablation) citation count model. SHAP summary plot from the top performing citation count model illustrating the contributions of the top 25 features towards the prediction. Each dot represents one sample for the features listed on the right. The x-axis represents the impact of that feature on the prediction, where dots on the right side of the vertical axis contribute to a positive prediction, i.e., higher citation count, and dots on the left side contribute to a negative prediction. Dots farther from the vertical axis indicate larger impact on the prediction. Red indicates a higher value of the feature (e.g., higher page count or higher number or many references). Blue values indicate lower values of the feature (e.g. low page count or few references). SHAP = Shapley Additive exPlanations.

Figure 3 is the SHAP plot illustrating the importance of the model. In the figure, the top 25 features are ranked from most important to least important.

**Figure 4:**
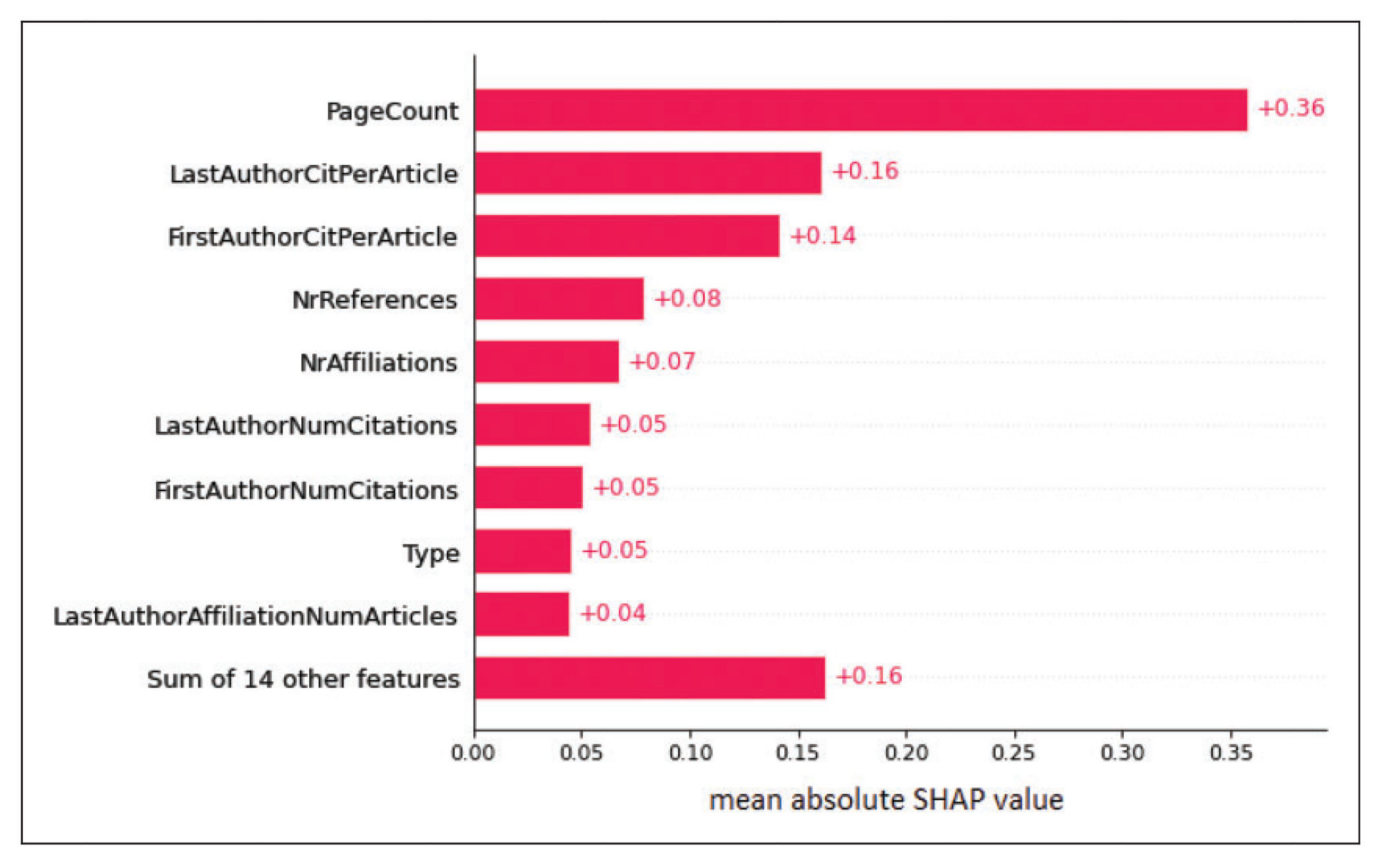
SHAP plot of the most important features in the citation count prediction model. SHAP plot of the relative importance of the top 10 features in the citation count prediction model. Mean absolute values are presented. This means that the features could be predicting both few and many citations. Note that page count is more important for the prediction than the sum of the 20 least important features.

Bibliometric features such as a high page count, high number of citations for the first and last author, high number of references included in the publication, many affiliations, high number of publications for the affiliations, and high author h-index predicted a high citation count. Publication type was the 8th most important feature, and reviews were generally predictive of many citations, whereas letters, editorials, and other short format publications were predictive of few citations. Figure 4 shows the 10 most important features and their relative impact on the predictions.

**Figure 5:**
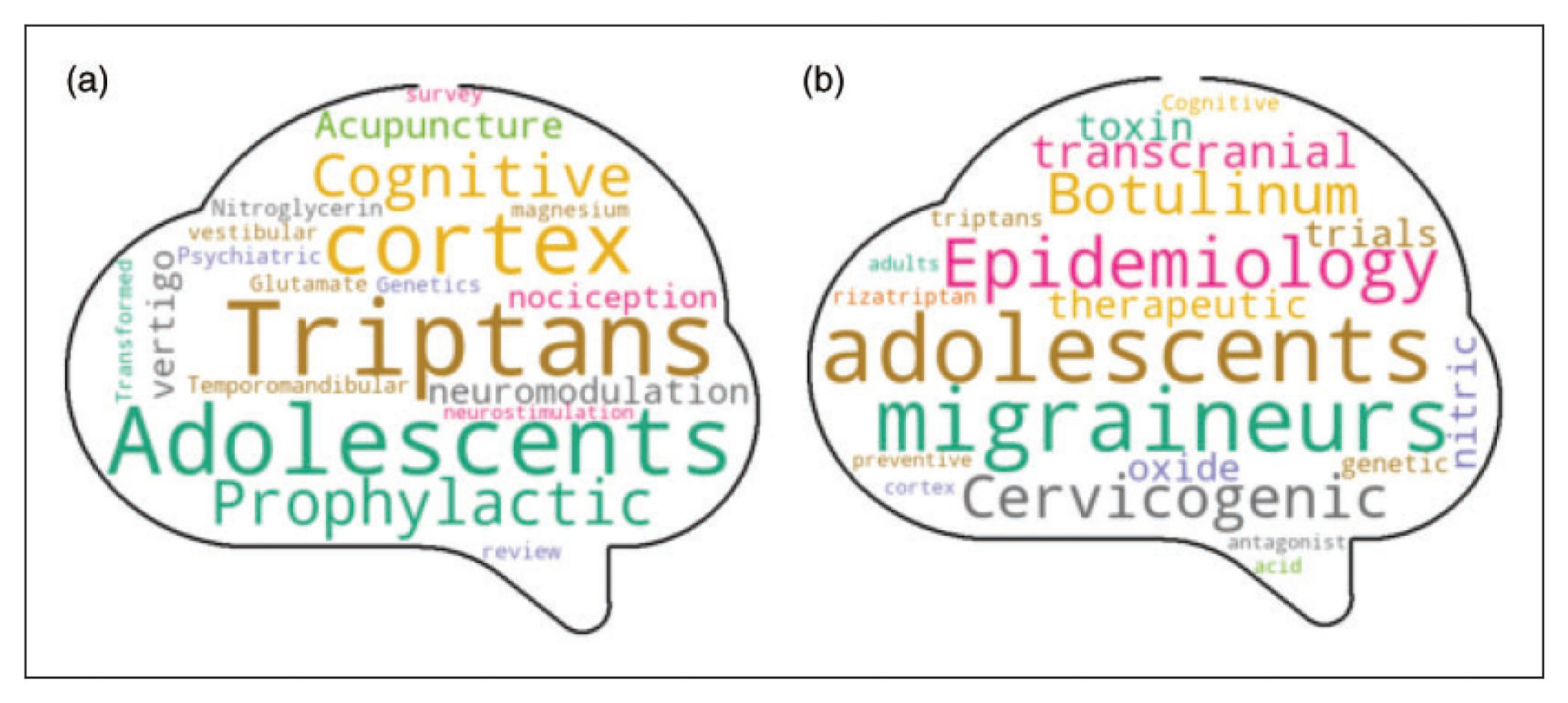
Word clouds of the keywords and titles of the most cited publications. Word clouds indicating the most frequent words used in the most cited articles of the three examined journals with respect to: (a) their keywords and (b) their titles. Words that are common in all publications regardless of citation counts have been excluded from the word clouds so that words unique to the ‘many citations’ classes are identified. Because research topics of interest change over time, words that are prominent in the word cloud does not alone predict high citation counts—a particular word must also be used at the correct time. Of note, COVID-19 is not appearing as the studies used for this analysis were published prior to 2018, while CGRP has rarely been used in the title and keywords of publications between the three journals inception and 2017. CGRP = calcitonin gene related peptide.

Figure 5 presents the word clouds outlining the most used words in the titles and keywords unique to the publications in the ‘many citations’ stratus.

### Translational impact modelling

**Figure 6:**
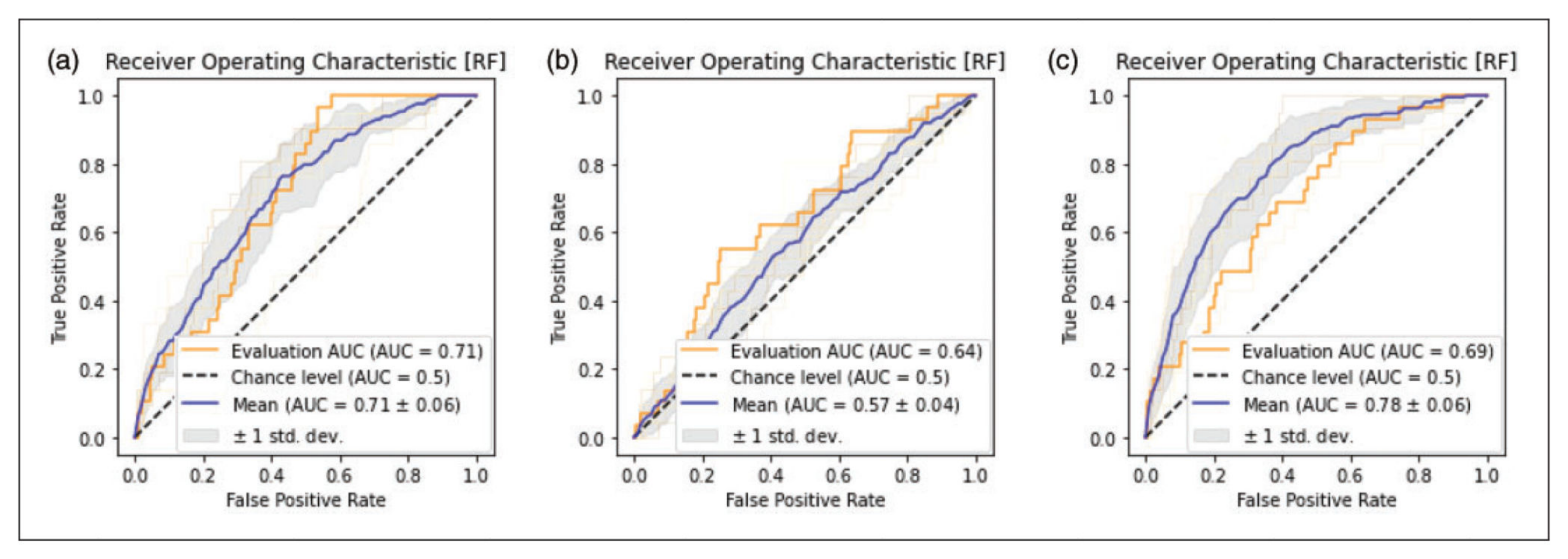
ROC plots of the optimized Random Forest model on translational impact prediction. ROC plots showing mean training performance (blue line) with 1 standard deviation (gray shaded area) and out-of-sample test set performance (orange line) for the translational impact models. (a) Model performance using both text-based and bibliometric features. (b) Model performance using only text-based features. (c) Model performance using only bibliometric features. The highest out-of-sample test set performance is achieved in the complete model (a). ROC = Receiver operating characteristics curve; AUC = Area under curve.

The training and validation results for the translation impact analysis are presented in Table 2 and the corresponding test set receiver operating characteristics curves are illustrated in Figure 6. The optimal modelling strategy for the translational impact was the Random Forest classifier, in part due to its excellence in handling heavily imbalanced datasets. The resampling strategies did not alter the balance of predictions. Out-of-sample performance for the Random Forest classifier was an AUC of 0.71 and a balanced accuracy of 0.59.

In the ablation analyses of the translational impact, results were generally more favorable when including both bibliometric features and text-based features (Table 2). Out-of-sample performance for the bibliometric data alone ablation analysis was an AUC of 0.69 and a balanced accuracy of 0.56. Out-of-sample performance for text-based data alone was an AUC of 0.64 and an accuracy of 0.55. The sensitivity analyses resulted in a test set AUC of 0.71 and an accuracy of 0.85 (Online Supplementary Figure 2).

**Figure 7:**
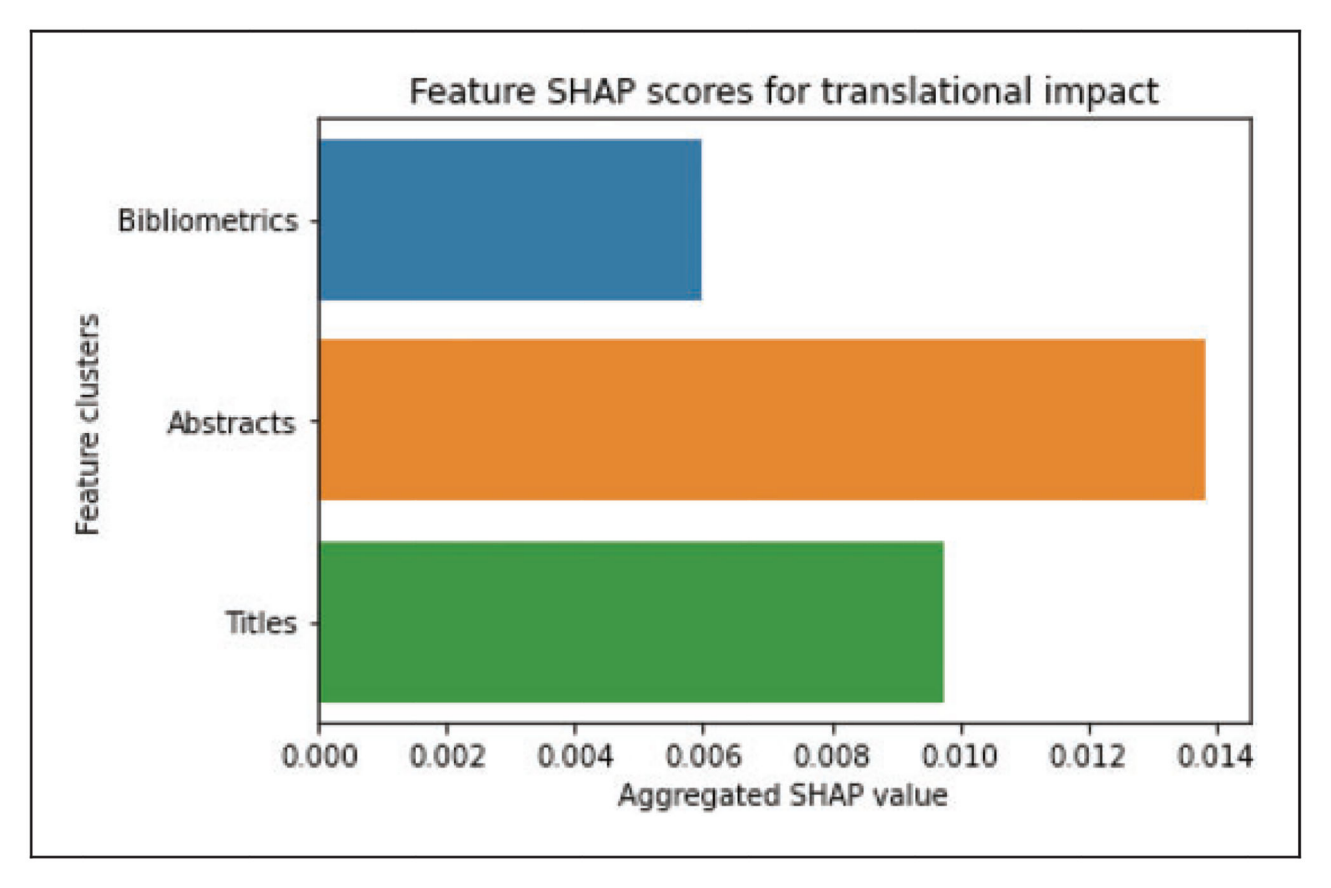
Contribution of input features to translational impact model. Aggregated absolute SHAP values for the bibliometric data, abstract derived text-based features and title derived text-based features on the translational impact model. Note that the combination of features from both the abstract and title contribute more to the prediction than the bibliometric data. SHAP = Shapley Additive exPlanations.

Figure 6 is a bar chart of the aggregated SHAP values for the bibliometric data, publication title features, and publication abstract features, respectively. Online Supplementary Figure 3 is a SHAP plot of the full translational impact model illustrating that the importance of the features is highly distributed and that title- and abstract-derived feature embeddings are equally important as the bibliometric data for the predictions (Figure 7).

## Discussion

Even though the literature on citation count prediction is vast there is not yet a headache domain-specific model for comparison. However, some studies illustrate benchmark performances in the general biomedical literature. Li and colleagues developed an exhaustive deep learning regression model of nearly 10 million biomedical papers which achieved an r^2^ of 0.78 for prediction of citation counts (22). The top performing model in our study achieved an AUC of 0.81. Nelson and colleagues created a field-wide deep learning model of more than 43.3 million published papers which accurately predicted inclusion in guide-line and policy document reference lists with an AUC of 0.92 and inclusion in patents with an AUC of 0.92 (9). In the latter study, predicting inclusion in guide-lines and policy documents were far superior when using publication content as compared to prediction from citation counts alone—serving as a solid argument that modeling of research content could guide the objective measurement of translational potential.

The predictive performance achieved in our study is close, but not as good as the benchmark models. Yet this is inevitable, given that model fidelity is correlated to data input size (23), which in our case is finitely limited by the available headache research volume. Nevertheless, the model presented here is highly specific for the headache research domain meaning that it, at present, offers an unrivaled performance as predictor for headache research citation count and translational impact.

The models developed in this paper can be relevant and applicable for both researchers, editors, and funders. As a researcher, navigating the scientific literature, especially unfamiliar domains, can be a daunting undertaking. Often, one looks to publications from renowned affiliations, published high-impact journals and with high citation counts to identify important works, not necessarily reflecting the most recent innovations. Editors must often make the decision to accept or reject a paper without knowledge of its future citation count or impact. Funders use classic research performance metrics such as citation counts to inform decisions (3). But citation counts as a sole measure of informing funding can be unreliable and irreplicable, and at times approach random (24), leaving room for improvement of how to choose, publish and fund research projects (25).

In our analysis of citation counts, bibliometric data appears to be more important than the contents of the paper for predictability. Relatively non-scientific and non-intuitive features such as page count, number of references, number of authors and number of author affiliations are identified as important predictors. Other studies have also indicated that such bibliometric data predicts citation counts. One study showed that the increasing length and number of authors seems to increase citation counts (26). Another review found that factors such as the length of the paper, the number of authors, and the number, prestige and variety of the references are associated with many citations (27).

The fact that increasing page count indicates more citations could be due to highly cited publications—such as position papers from neurological societies, guidelines, classifications and reviews—tending to be longer, and that lengthy papers may appear more complex and methodologically robust thus gaining more attention and citations. Reviews also predict many citations as compared to original publications, whereas the short-format publications predict fewer citations (Figure 2). Not surprisingly, the number of articles, number of citations and h-index of the first and last author are also highly predictive of citation counts. This is well-established in the general bibliometric literature (27). A bibliometric study from 2017 sought to identify the 100 top cited headache related publications (28) and demonstrated that large proportions of the citations seem to accumulate among relatively small groups of journals, authors and affiliation. More than half of the 100 papers were published in one of the three journals *Neurology, Cephalalgia* or *Headache*, five institutions represented nearly a third of the papers, and five authors were first authors on nearly a fourth of the papers. Of course, such accumulation of acknowledgement is warranted by the respective institutions and authors scientific excellence. Still, researchers, editors and policy-makers alike must keep in mind that “reasons for citation” may be the result of a myriad of both scientific and non-scientific reasons (5,27). Finally, a strength of the machine learning models lies in their ability to identify patterns across a large number of predictors. Even though a single feature, such as journal impact factor, may seem unimportant, it is only in conjunction with many other features that it has the capacity to inform the prediction.

The notion that bibliometric data alone predict the citation count, and thus indicates the importance of a scientific work, must be considered with caution (29). Our translational impact modelling demonstrates that research content is just as important as bibliometric data in predicting translational potential and clinical importance. In addition, the importance of the features in the translational impact model is highly distributed meaning that all the features are necessary for optimal model performance (Online Supplementary Figure 1). On the contrary, the citation count model uses primarily the top 10–15 features to inform the prediction. This discrepancy in feature dimensionality, i.e. the number of features needed for optimal performance, is important. Benchmark predictive models clearly show that it is the models that include the widest array of both bibliometric and publication content input data that yields the highest fidelity in identifying the clinical importance of a scientific work (9).

One must also keep in mind that many of the author related features (h-index, number of citations and articles) were captured at the timepoint when this study was conducted. This is a methodological weakness; however, we argue that our findings still are valid for several reasons. Firstly, the citation count models include personal author information not influenced by this “time-bias” such as affiliations. Secondly, other bibliometric meta-data features seem to be just as important for the citation count predictions, validating the model. This is also confirmed by the sensitivity analysis showing only slightly poorer performance. Thirdly, the ablated citation count model using only text-based features has a moderate predictive accuracy further validating the precision of the model regardless of a potential “time-bias” from author citation counts at the time of data collection. Finally, the translational impact models are not influenced as the outcome (inclusion in a guideline or policy document) is not decided by the citation count. The same weakness is true for the journal impact factor, also captured at the timepoint of analyses. Future models should attempt to gather historical publication metrics and impact factors to improve model precision.

A few other limitations must also be kept in mind when interpreting the results of this study. First, the models are based on retrospective data. This means that it learns the patterns of the publishers, journals, researchers, and guideline- and policymakers in identifying citations counts and relevant clinical impacts. If, for example, a new medication, not available in the training data literature, is introduced to the model, its potential importance will not be intuitively captured by the model, and it will likely be misclassified. Second, the included journals publish a combination of basic, translational, and clinical research, but the two former types are underrepresented in guidelines and policy documents. One must therefore suspect that the importance of basic and translational research is undervalued in the translational impact modelling. A possible future prospect for identifying the importance of basic and translational research could be modelling of inclusion in both technical and pharmacological patents. Likewise, the translational impact does not incorporate inclusion in headache and neurology specific guidelines, and communication to the public. Third, several factors that intuitively could influence the predictions were not included in the models: presentation of the paper at conferences, funding sources, being part of a special collection, the presence of multinational authors, if the manuscript had previously been submitted to other journals, or a publication stemming from the boards of neurological of headache societies. Such factors should be incorporated in future models. Fourth, a substantial portion of publications had missing data and were excluded from the analyses which could impact the results—on the other hand, a choice to impute data could also lead to biased results. Finally, the input data was limited to three headache-related journals, yet many milestone papers in headache research have been published in high-impact general journals. We argue that it is unlikely that this would influence the accuracy of the citation count models as these publications represent the extreme end of citation counts which is easier to predict, and most likely adhere similar methodology and reporting standard as the papers included in the model.

## Conclusion

In this study we demonstrate that machine learning models may predict the citation count of headache research papers with good accuracy. Bibliometric data such as high page count, many authors, many references, first and last authors with many citations and high author h-index seems to be among the most important predictors for high citation counts. On the other hand, complex models of both bibliometric data and publication content identify the translational impact of headache scientific works. We argue that citation counts alone as a metric of the importance of a scientific work must be used with caution, whereas models incorporating publication content could be used to guide researchers, editors, and funders in identifying the most relevant and potentially impactful scientific works.

## Supplementary Material

Supplementary material

## Figures and Tables

**Table 1:** Performance of different modelling strategies in predicting citation counts.

Model	NLP strategy for citation count prediction		
	Doc2Vec	SpaCy	BioBERT
Ada Boosting	0.63 (SD = 0.019); 0.64	0.68 (SD = 0.025); 0.66	0.66 (SD = 0.025); 0.66
Support Vector Machine	0.63 (SD = 0.023); 0.65	0.67 (SD = 0.027); 0.65	0.63 (SD = 0.018); 0.63
Random Forest	0.65 (SD = 0.021); 0.66	0.65 (SD = 0.021); 0.66	0.66 (SD = 0.020); 0.66
Naïve Bayes	0.65 (SD = 0.021); 0.63	0.62 (SD = 0.027); 0.62	0.64 (SD = 0.021); 0.63
Decision Tree	0.53 (SD = 0.024); 0.54	0.57 (SD = 0.014); 0.55	0.55 (SD = 0.021); 0.55
K-Nearest Neighbor	0.63 (SD = 0.024); 0.64	0.62 (SD = 0.025); 0.63	0.63 (SD = 0.022); 0.64
Gradient Boosting	0.70 (SD = 0.025); 0.71	0.67 (SD = 0.022); 0.69	0.69 (SD = 0.008); 0.70
Multilayer Perceptron	0.69 (SD = 0.016); 0.69	0.66 (SD = 0.025); 0.66	0.69 (SD = 0.025); 0.69
Deep Learning	0.51 (SD = 0.330); 0.52	0.53 (SD = 0.045); 0.53	0.58 (SD = 0.019); 0.58

Performances for the different machine learning classifiers and NLP strategies before hyperparameter optimization and feature selection scored with 10-fold mean cross-validated AUC with standard deviation on the training set and AUC on the validation set. AUCs are calculated as the micro-averaged One-vs-Rest. The choice of model for further analyses was based on validation set performance. Gradient Boosting with Doc2Vec NLP strategy showed best performance during training and validation and was used for out-of-sample test set scoring. NLP = Natural Language Processing; AUC = Area Under the receiver operating characteristics Curve; SD = Standard Deviation.

**Table 2:** Performance of different modelling strategies in predicting translational impact

Model	Translational impact prediction		
	Bibliometric features	Text-based features	All features
Ada Boosting	0.70 (SD = 0.045); 0.70	0.50 (SD = 0.045); 0.50	0.5l (SD = 0.010); 0.5l
Support Vector Machine	0.70 (SD = 0.063); 0.70	0.55 (SD = 0.055); 0.56	0.65 (SD = 0.063); 0.67
Random Forest	0.70 (SD = 0.059); 0.70	0.57 (SD = 0.043); 0.60	0.7l (SD = 0.059); 0.72
Naïve Bayes	0.66 (SD = 0.07l); 0.67	0.55 (SD = 0.043); 0.56	0.60 (SD = 0.074); 0.60
Decision Tree	0.52 (SD = 0.023); 0.5l	0.50 (SD = 0.024); 0.50	0.5l (SD = 0.0l6);0.52
K-Nearest Neighbor	0.52 (SD = 0.045); 0.5l	0.50 (SD = 0.036); 0.50	0.49 (SD = 0.020); 0.50
Gradient Boosting	0.63 (SD = 0.164); 0.65	0.63 (SD = 0.164); 0.65	0.64 (SD = 0.l45); 0.64
Multilayer Perceptron	0.47 (SD = 0.045); 0.48	0.48 (SD = 0.056); 0.49	0.52 (SD = 0.064); 0.56
Deep Learning	0.60 (SD = 0.030); 0.60	0.53 (SD = 0.051); 0.54	0.53 (SD = 0.053); 0.53

Performances for the different machine learning classifiers with different sets of input features scored with 10-fold mean cross-validated AUC with standard deviation on the training set and AUC on the validation set. The columns titled “Bibliometric features” and “Text-based features” refer to the two ablation analyses. The rightmost column shows the results of the complete translational impact model including both bibliometric and text-based features. The top performing model in validation was Random Forest which subsequently was used on the test set. AUC = Area Under the receiver operating characteristics Curve; SD = Standard Deviation.

## Clinical implications

- Machine learning can be used to predict the citation counts of headache papers and predict which papers will be included in guideline and policy documents.
- High citation counts are mainly predicted by bibliometric data such as high page count, many references, high author citation counts, and high author h-index.
- The future impact of a headache research paper is predicted by the contents of its title, abstract and keywords.
