# Supplementary material for: What predicts citation counts and translational impact in headache research? A machine learning analysis
Source: Cephalalgia. Author manuscript; Available in PMC 2025 Sep 18. (PMC7618138; doi:10.1177/03331024241251488)
Supplement: Supplementary material [file EMS208348-supplement-Supplementary_material.pdf]

Supplementary material

Supplementary table 1: Bibliometric and text-based features used for predictions.

Variable name	Description
FirstAuthorNumArticles	The number of articles written by the 1st author
FirstAuthorNumCitations	The number of 1st author's citations
FirstAuthorHindex	The h-index metric of the 1st author
FirstAuthorCitPerArticle	The citations per article metric of the 1st author
FirstAuthorAffiliationMeanHindex	The mean h-index metric of the authors coming from 1st author's affiliation.
LastAuthorNumArticles	The number of articles written by the last author
LastAuthorNumCitations	The number of last author's citations
LastAuthorHindex	The number of articles written by the 1st author
LastAuthorCitPerArticle	The citations per article metric of the last author
LastAuthorAffiliationMeanHindex	The mean h-index metric of the authors coming from last author's affiliation.
LastAuthorAffiliationNumArticles	The number of articles sourcing by the last author's affiliation
Year	Year of the publication
Volume	Journal volume of the publication
PageCount	The total number of pages of the paper
NrAffiliations	The number of different affiliations of the authors of the paper
NrReferences	The number of references in the paper
OpenAccess	Whether the publication is an open access manuscript or not
NrAuthors	The number of different authors of the paper
Journal	The journal that the paper is published
PubType	The type of publication (article, review, note, letter, editorial, erratum, short survey or conference paper)
JournalImpactFactor	The journal overall impact factor
JournalImpactFactorNoSelfCit	The journal overall impact factor not considering self-citations
Journal5YearImpactFactor	The journal 5-year impact factor
JCI	The journal citation indicator
ImmediacyIndex	The immediacy index of the journal
CiteScore	The Cite Score of the journal
SJR	The SCImago Journal Rank of the journal
SNIP	The Source Normalized Impact per Paper of the journal
Title Doc2Vec	Title converted to 300-dimensional vector

Abstract Doc2Vec	Abstract converted to 300-dimensional vector
Keywords Doc2Vec	Keywords converted to 300-dimensional vector

Features included in the predictive models. Only the bibliometric features (except from FirstAuthorAffiliationMeanHindex, LastAuthorAffiliationMeanHindex, Year and Volume) were used for the top performing citation count model. The same bibliometric features along with the text-based features (the last three rows of the table) were used for the top performing translational impact model.

JCI: The Journal Citation Indicator is the average of the division of the actual count of citing items by the expected citation rate for documents with the same document type, year of publication and subject area.

ImmediacyIndex: The Immediacy Index is the count of citations in the current year to the journal that reference content in this same year. Journals that have a consistently high Immediacy Index attract citations rapidly.

CiteScore: Cite Score counts the citations of a journal, received in a 3-year period, to articles, reviews, conference papers, book chapters and data papers published within the 3-year period, and divides this by the number of publications published within the same period.

SJR: The SCImago Journal Rank (SJR) is a measure that considers the number of citations received by a scholarly journal as well as the prestige of the journals where the citations come from.

SNIP: Source-Normalized Impact per Paper (SNIP) measures the impact of a paper within a subject field.

Supplementary table 2: Publications dataset

	**Cephalalgia**	**J. Headache & Pain**	**Headache**	**Total**
Original papers	3081	2588	812	6481
Conference Papers	153	1	35	189
Editorials	145	34	67	246
Erratum	1	0	2	3
Letters	216	49	275	540
Notes	31	19	86	136
Reviews	390	111	432	933
Short Surveys	19	2	51	72
Total	4036	2804	1760	8600

Overview of publication types captured through Scopus from the three included headache-oriented journals.

Supplementary table 3: Grid searched and final hyperparameters for both citation count model and translational impact model.

CITATION COUNT PREDICTION MODEL			
HYPERPARAMETERS	DEFAULT VALUES	SEARCH GRID	FINAL VALUES
learning_rate	0.1	[0.005, 0.01, 0.05, 0.1, 0.2, 0.3]	0.1
loss	log_loss	[log_loss, exponential]	log_loss
n_estimators	100	[50, 500]	100
criterion	friedman_mse	[friedman_mse, squared_error]	squared_error
max_depth	3	[0, 30]	20
max_features	None	[sqrt, log2, None]	None
min_samples_leaf	1	[1, 4]	1
min_samples_split	2	[2, 10]	2
TRANSLATIONAL IMPACT PREDICTION MODEL			
HYPERPARAMETERS	DEFAULT VALUES	SEARCH GRID	FINAL VALUES
bootstrap	True	[True, False]	True
n_estimators	100	[50, 500]	100
min_samples_leaf	1	[1, 4]	2
min_samples_split	2	[2, 10]	2
max_depth	None	[0, 30]	8
max_features	sqrt	[sqrt, log2, None]	sqrt
class_weight	None	[None, balanced, balanced_subsample]	balanced_subsample
criterion	gini	[gini, entropy, log_loss]	entropy

Supplementary Figure 1: Sensitivity analysis on citation count prediction

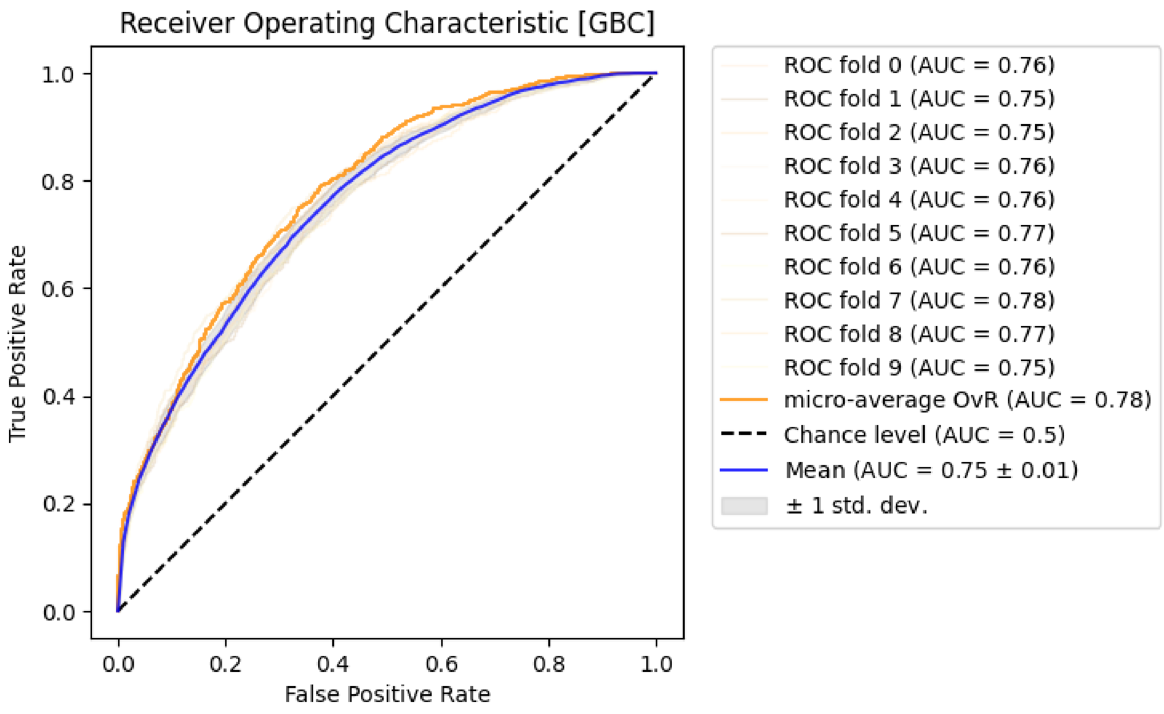

ROC plot of the optimized Gradient Boosting model on citation count prediction when removing the features first author citation count, first author cites per article, first author h-index, last author citation count, last author cites per article, last author h-index. The ROC plots shows mean training performance (blue line) with 1 standard deviation (gray shaded area) and out-of-sample test set performance (orange line). AUCs are calculated as the micro-averaged One-vs-Rest. Note that the AUC and accuracy are only slightly poorer as compared to the primary analysis.

ROC = Receiver operating characteristics curve; AUC = Area under curve; OvR = One-vs-Rest

Supplementary Figure 2: Sensitivity analysis on translational impact prediction

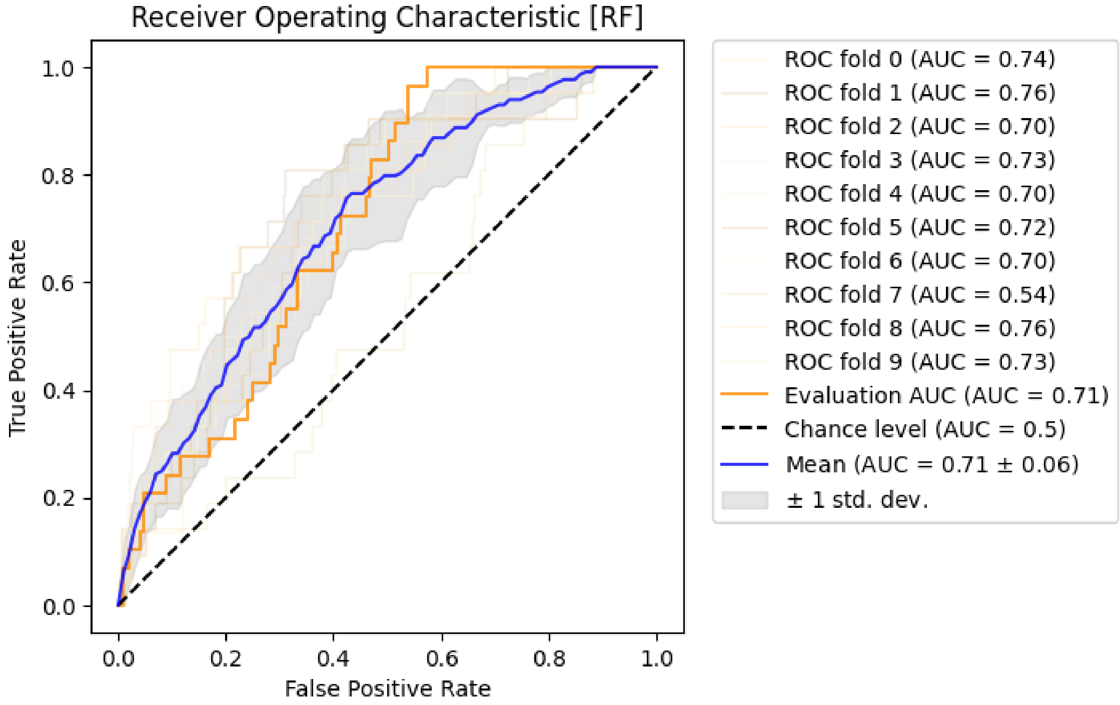

ROC plot of the optimized Random Forest model on translational impact prediction when removing the features first author citation count, first author cites per article, first author h-index, last author citation count, last author cites per article, last author h-index. The ROC plots shows mean training performance (blue line) with 1 standard deviation (gray shaded area) and out-of-sample test set performance (orange line). AUCs are calculated as the micro-averaged One-vs-Rest. Note that both the AUC and accuracy are improved as compared to the primary analyses.

ROC = Receiver operating characteristics curve; AUC = Area under curve; OvR = One-vs-Rest

Supplementary Figure 3: SHAP summary plot for top translational impact model

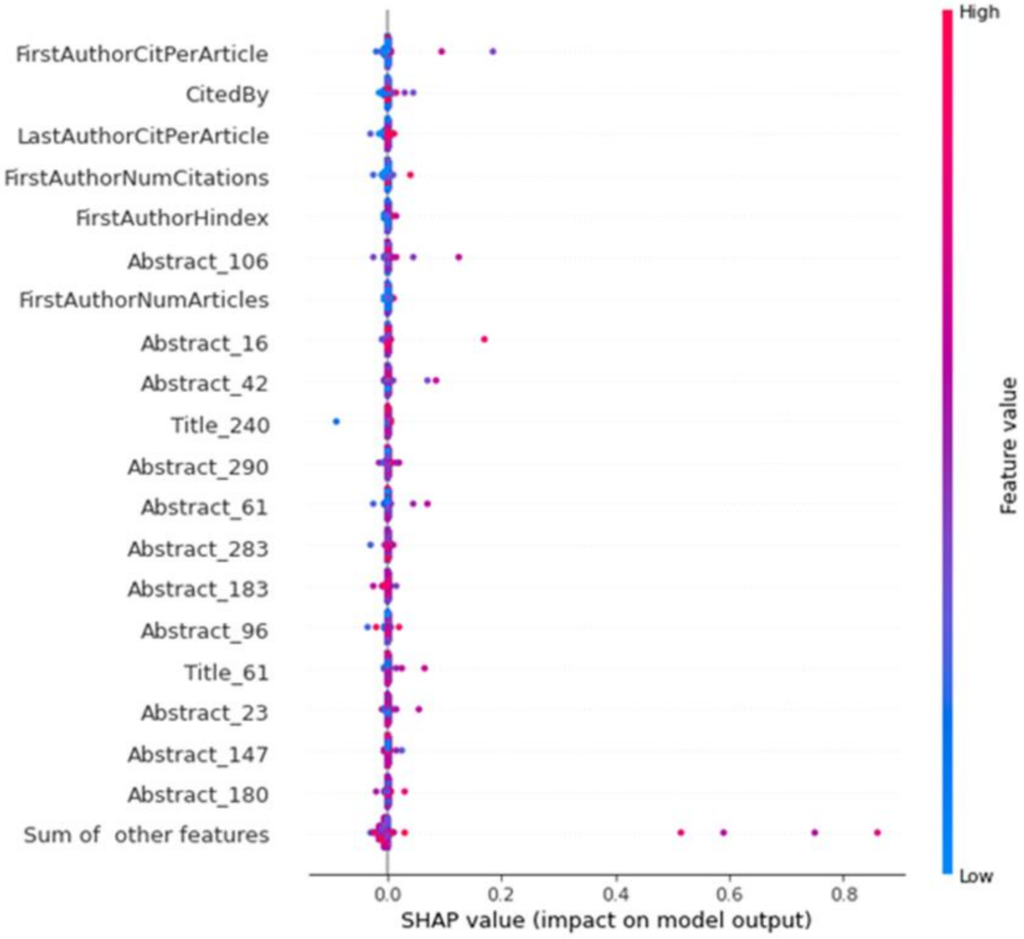

SHAP plot derived from the top performing translational impact model illustrates highly distributed feature importances. Most features are weighted near equally supporting the argument that the whole text-based embedding spaces need to be included in the model for optimal performance. Moreover 68% of the top 20 features are text-based.

SHAP = Shapley Additive exPlanations.

Supplementary Figure 4: SHAP summary plot the citation count model which uses both text-based and bibliometric features

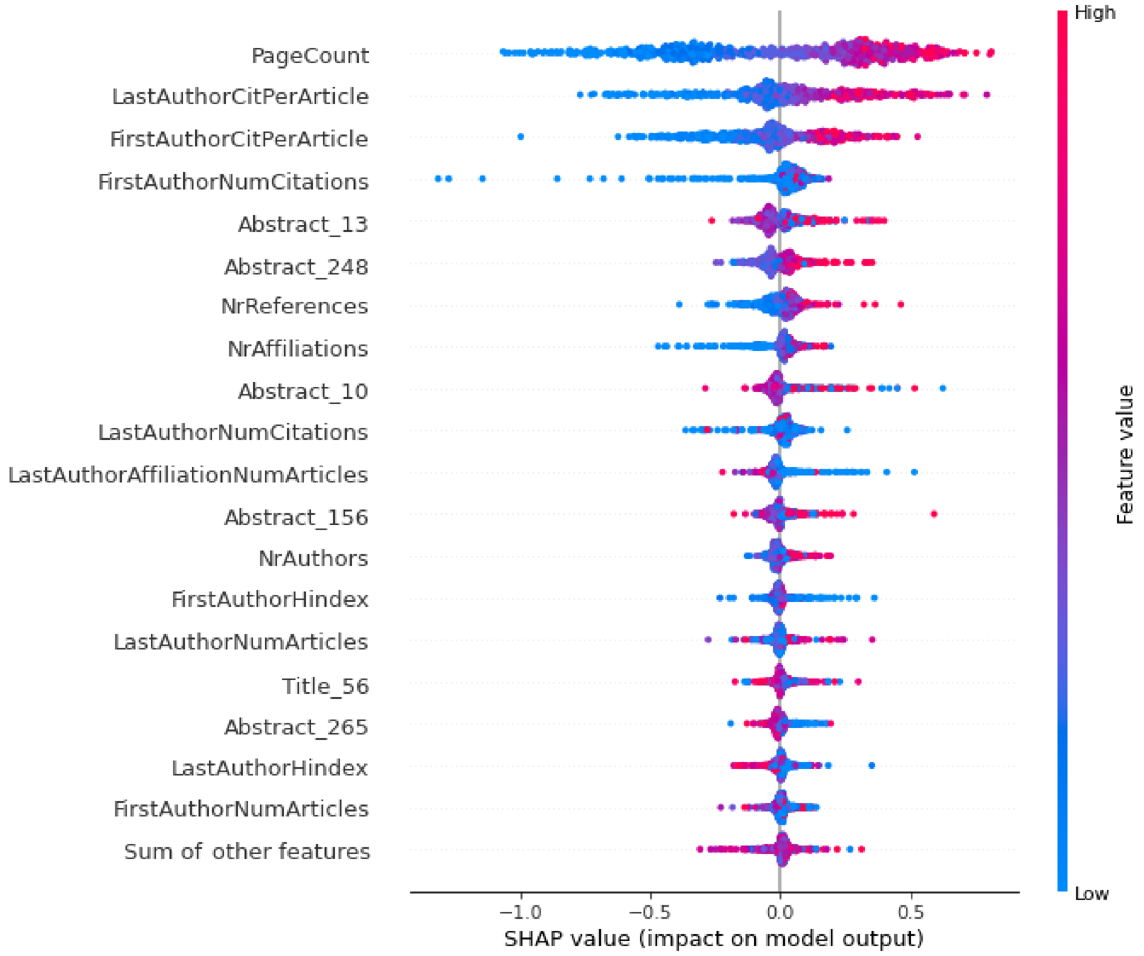

SHAP plot derived from the citation count prediction model that uses both text-based and bibliometric features illustrates highly feature importances for the bibliometric features. Most text-based features do not significantly contribute towards the final prediction. In fact, only 30% of the top 20 features are text-based.

SHAP = Shapley Additive exPlanations.
